# Supplementary material for: Responses of Aerial and Belowground Parts of Different Potato (Solanum tuberosum L.) Cultivars to Heat Stress
Source: Plants (Basel). 2023 Feb 12;12(4):818. doi: 10.3390/plants12040818 (PMC9964869; doi:10.3390/plants12040818)
Supplement: Supplementary file 1 [file plants-12-00818-s001.zip › plants-2174706-supplementary.pdf]

Table S1. Primers used in this work, related to the SYBR Green I method.

No.	Primers	Sequences
1	*StSP6A_F*	5′-GACGATCTTCGCAACTTTTACA
	StSP6A_R	5′-CCTCAAGTTAGGGTCGCTTG
2	*StBEL5_F*	5′-GGACCATCATCTAAGGCAACAACG
	StBEL5_R	5′-AAGCACGAAGGACAGAGACAGC
3	*StGA2ox1_F*	5′-ATCCACCATGTCCAGAGTTG
	StGA2ox1_R	5′-GGATCAGGTGGGACTGAGAT
4	*StPOTLX-1_F*	5′-AGAGGAGATGGAACTGGAAAGCG
	StPOTLX-1_R	5′-GAGGATACGGGTAGTCAGCAGAAC
5	*StAGPase_F*	5′-TCTGTGGTTGTTTGAGGAGCATAC
	StAGPase_R	5′-GCGGCAACGGTAATATCAGCATC
6	*StTFL1_F*	5′-CTAGGGTTGAAGTTCATGGTGGTG
	StTFL1_R	5′-TCTGTAGTGCCTGGAATGTCTGTG
7	*StCOL1_F*	5′-CGCATACATGCAGCAAGTCTC
	StCOL1_R	5′-GAATGGGCATAATTGGGACACG
8	*StSP5G_F*	5′-CAACTCCGAGCAATCCTTACCAC
	StSP5G_R	5′-TTGTAGGTCTTGGGCACTCATAAG
9	*StPHYB_F*	5′-TGCGATGGTGCTGCTCTATACTAC
	StPHYB_R	5′-GCCAATAACCACTCCACAATGTCC
10	*StBEL29_F*	5′-CGGTAACAACAACAACAACGAGTC
	StBEL29_R	5′-CCTTGCTGCTGCTGATGATGAG
11	*StSUT4_F*	5′-GCTTCTCTTTGCCGCCCTTG
	StSUT4_R	5′-AACCTTGTCCTGAACCAGCATTAC
12	*StTOC1_F*	5′-TGAGGGGACAGTTTGTAAGGAAGG
	StTOC1_R	5′-TTCAACATCGTCATTGCCTGCTG
13	*StEF1α_F*	5′-ATTGGAAACGGATATGCTCCA
	StEF1α_R	5′-TCCTTACCTGAACGCCTGTCA

The primers used were designed using allele ID 6. F: forward primer; R: reverse primer.

Table S2. Reads and reference genome comparison.

Materials	Total mapped	Materials	Total mapped	Materials	Total mapped	Materials	Total mapped
D187EN L-1	86.53%	D187EN T-1	90.13%	Qs9EN L-1	89.87%	Qs9EN T-1	86.04%
D187EN L-2	86.42%	D187EN T-2	91.29%	Qs9EN L-2	89.96%	Qs9EN T-2	85.09%
D187EN L-3	85.98%	D187EN T-3	89.62%	Qs9EN L-3	90.03%	Qs9EN T-3	85.30%
D187UH L-1	87.46%	D187UH T-1	88.83%	Qs9UH L-1	90.68%	Qs9UH T-1	85.18%
D187UH L-2	86.64%	D187UH T-2	88.46%	Qs9UH L-2	89.81%	Qs9UH T-2	84.82%
D187UH L-3	86.28%	D187UH T-3	87.63%	Qs9UH L-3	89.68%	Qs9UH T-3	83.57%
D187EH L-1	86.23%	D187EH T-1	88.25%	Qs9EH L-1	89.29%	Qs9EH T-1	84.69%
D187EH L-2	85.14%	D187EH T-2	87.30%	Qs9EH L-2	88.44%	Qs9EH T-2	83.03%
D187EH L-3	86.51%	D187EH T-3	88.18%	Qs9EH L-3	89.26%	Qs9EH T-3	84.65%
D187AH L-1	85.98%	D187AH T-1	89.42%	Qs9AH L-1	88.75%	Qs9AH T-1	85.83%
D187AH L-2	85.68%	D187AH T-2	88.78%	Qs9AH L-2	88.27%	Qs9AH T-2	83.06%
D187AH L-3	87.13%	D187AH T-3	90.01%	Qs9AH L-3	89.25%	Qs9AH T-3	85.34%

L: leaf, T: tuber. 1, 2 and 3 represent one biological repetition of the same treatment.

Table S3. The 12 genes reported to regulate the tuberization of potato plants.

No.	Gene name	Gene ID	Effect on tuberization	Reference
1	*StSP6A*	Soltu.DM.05G026370	Activator	Control of flowering and storage organ formation in potato by FLOWERING LOCUS T
2	*StBEL5*	Soltu.DM.06G029500	Activator	Dynamics of a mobile RNA of potato involved in a long-distance signaling pathway
3	*StGA2ox1*	Soltu.DM.02G013470	Activator	StGA2ox1 is induced prior to stolon swelling and controls GA levels during potato tuber development
4	*StPOTLX*	Soltu.DM.08G005480	Activator	Lipoxygenase is involved in the control of potato tuber development
5	*StAGPase*	Soltu.DM.07G022290	Activator	Carbon partitioning mechanisms in potato under drought stress
6	*StTFL1*	Soltu.DM.06G029780	Activator	Cloning and characterization of a potato TFL1 gene involved in tuberization regulation
7	*StCOL1*	Soltu.DM.02G030260	Repressor	Potato StCONSTANS-like1 Suppresses Storage Organ Formation by Directly Activating the FT-like StSP5G Repressor
8	*StSP5G*	Soltu.DM.11G004050	Repressor	Naturally occurring allele diversity allows potato cultivation in northern latitudes
9	*StPHYB*	Soltu.DM.01G019510	Repressor	Phytochrome B affects the levels of a graft-transmissible signal involved in tuberization
10	*StBEL29*	Soltu.DM.01G002850	Repressor	Multiple Mobile mRNA Signals Regulate Tuber Development in Potato
11	*StSUT4*	Soltu.DM.11G010180	Repressor	Sucrose transporter StSUT4 from potato affects flowering, tuberization, and shade avoidance response
12	*StTOC1*	Soltu.DM.06G025760	Repressor	Identification of TIMING OF CAB EXPRESSION 1 as a temperature-sensitive negative regulator of tuberization in potato

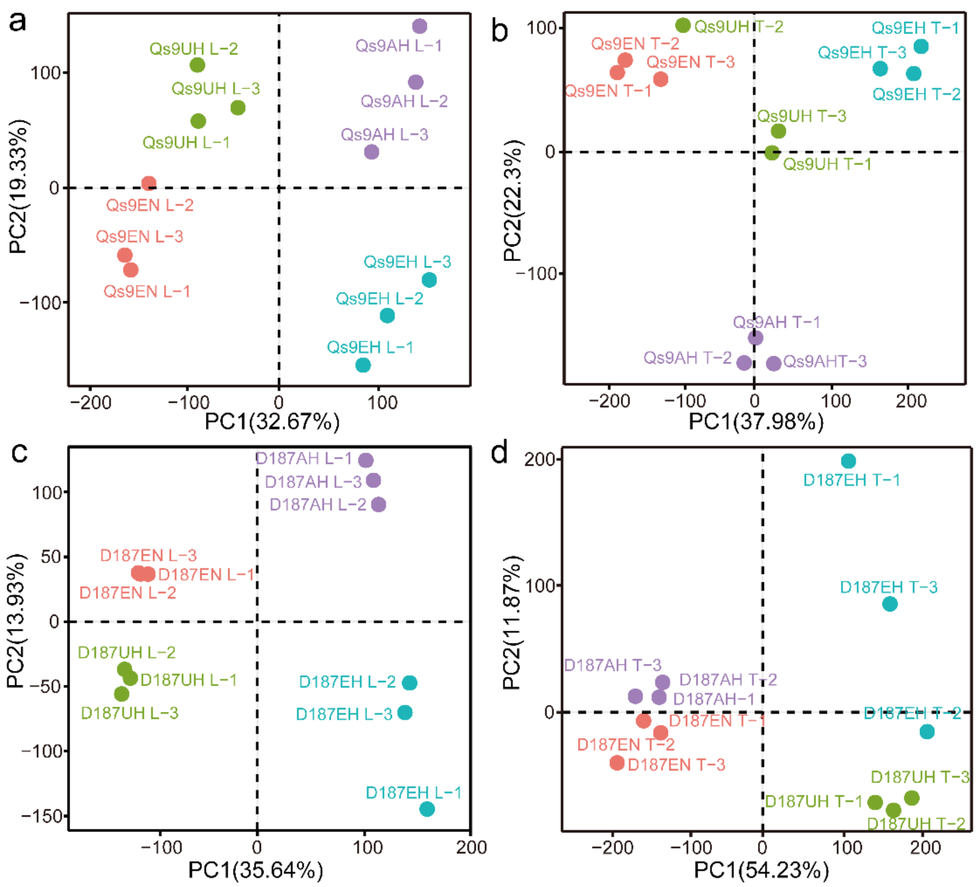

Figure S1. A PCA plot of transcriptome data. L: leaf, T: tuber. 1, 2 and 3 represent one biological repetition of the same treatment. Three biological replicates are shown in the same colour.

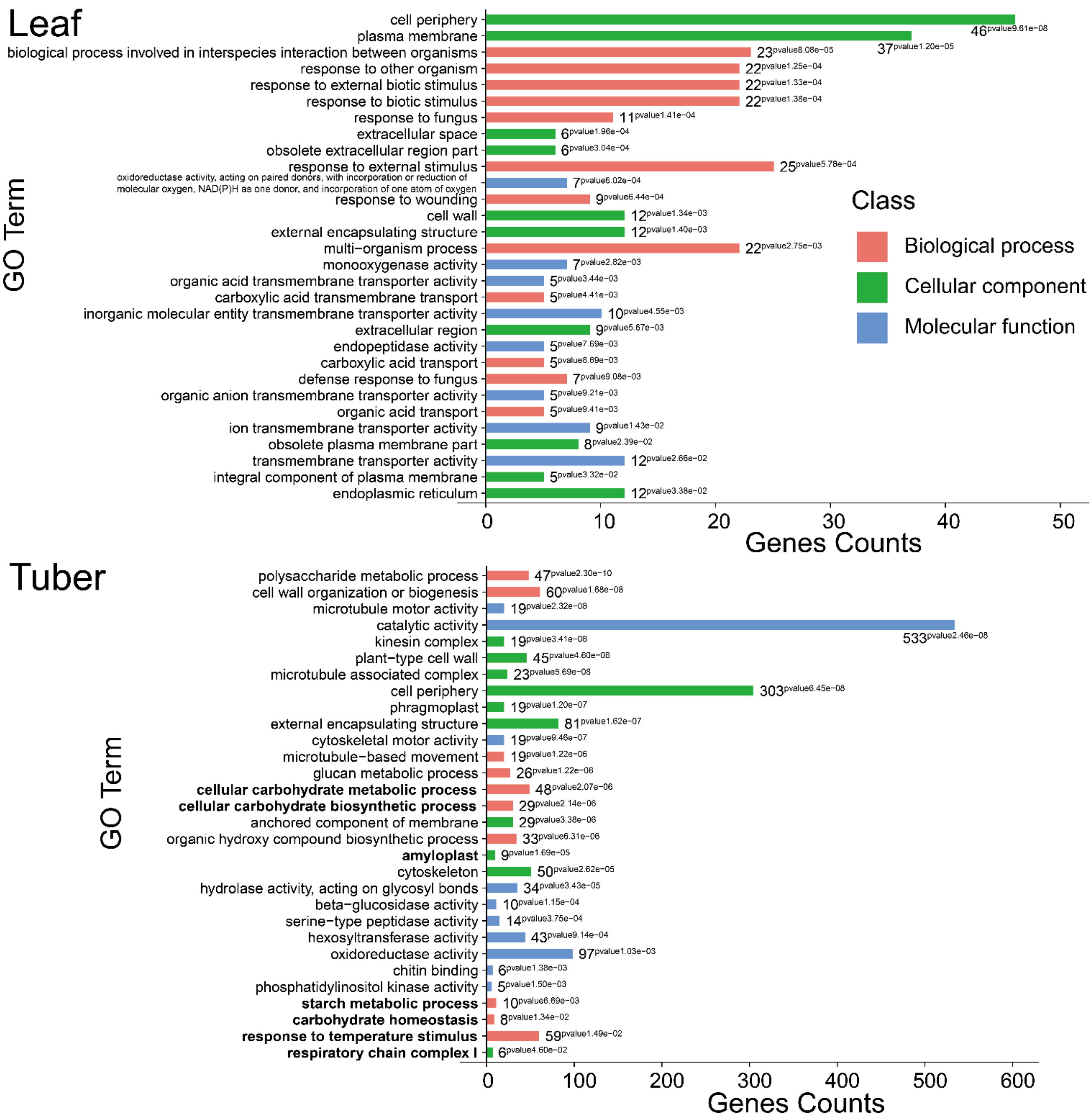

Figure S2. GO analysis of the DEGs in the leaves and tubers of the Qs9 plants under the heat-stress treatment of the different plant parts (affecting tuberization).

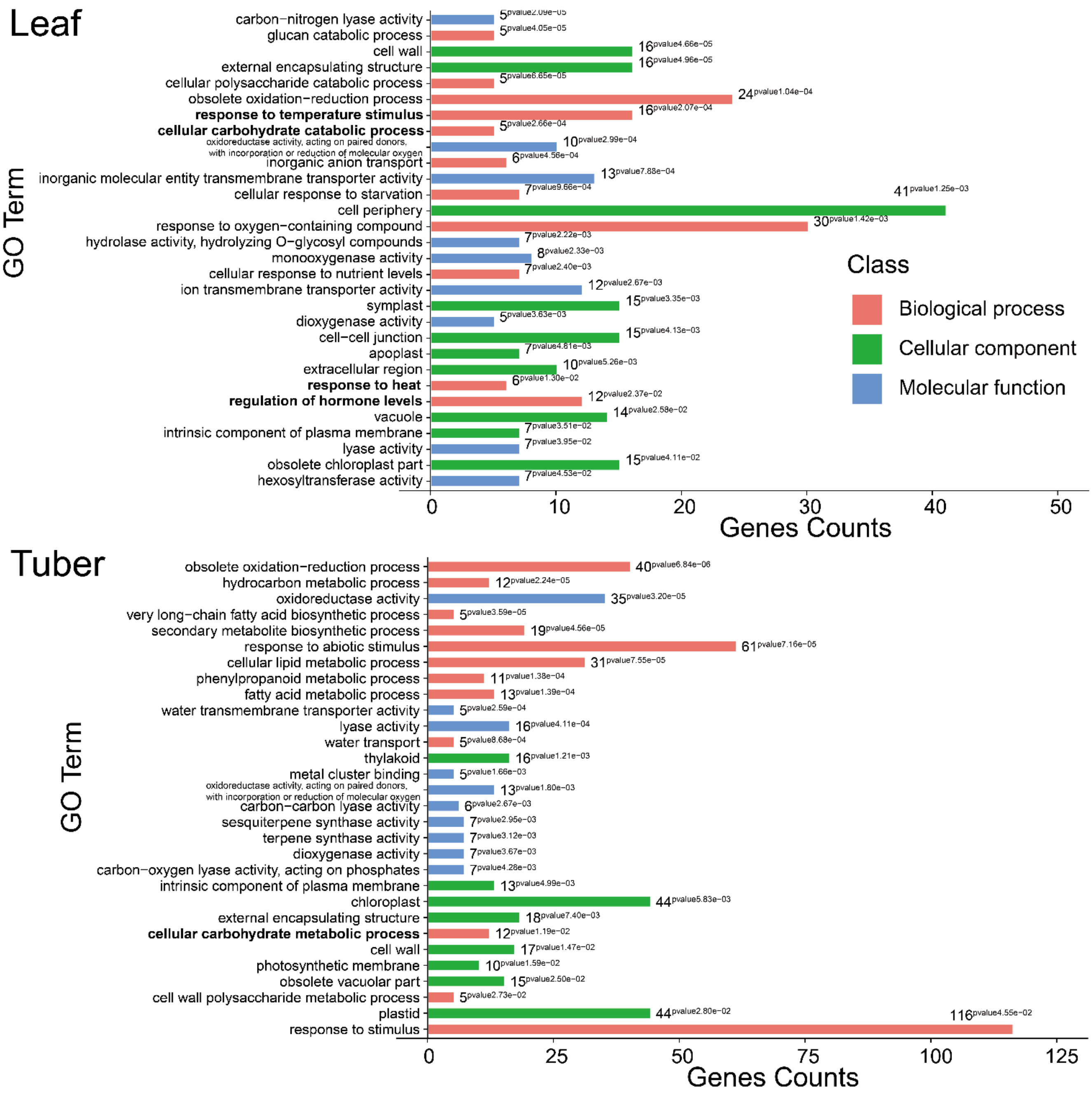

Figure S3. GO analysis of the DEGs in the leaves and tubers of the D187 plants under the heat-stress treatment of different plant parts (affecting tuberization).

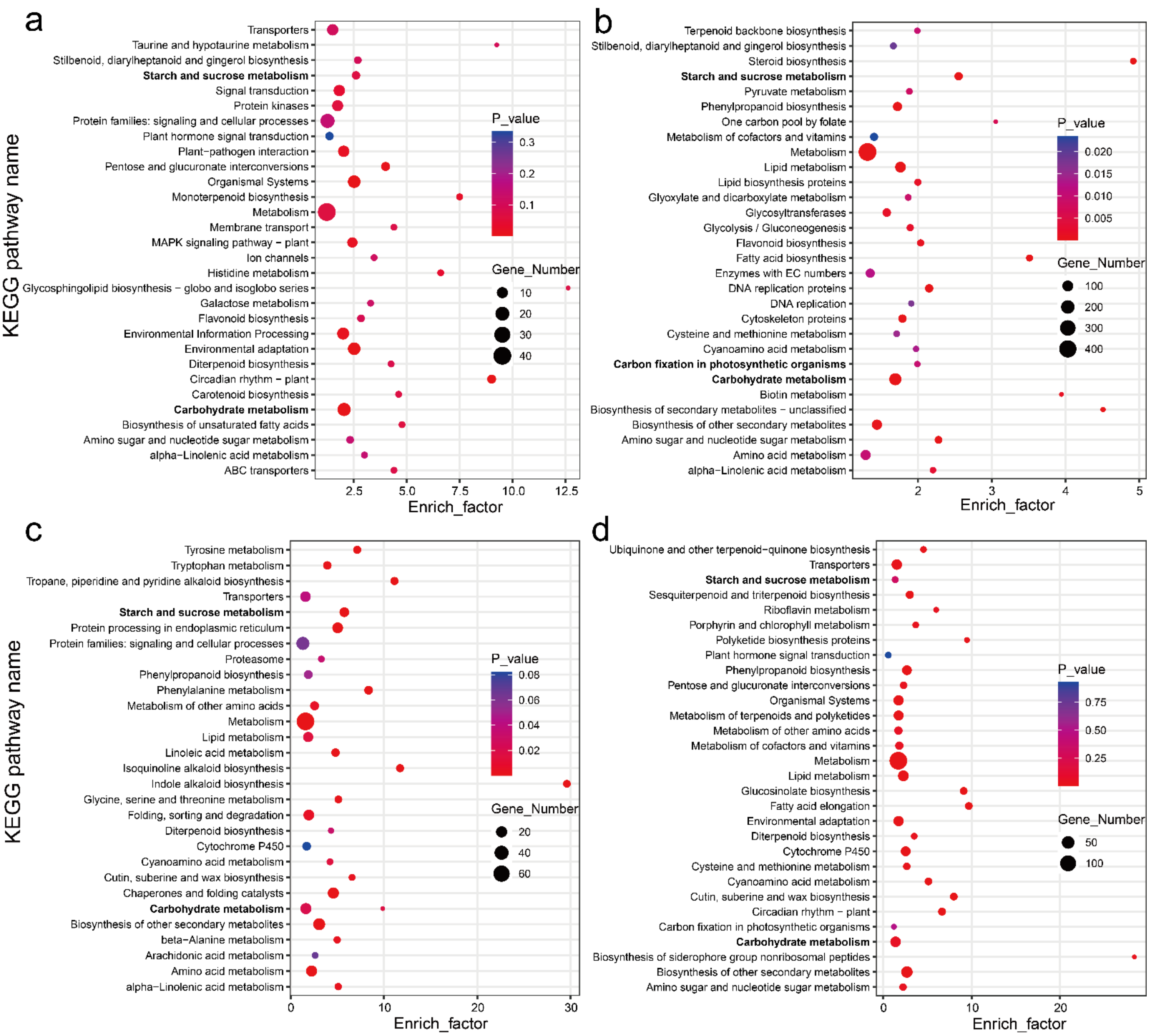

Figure S4. KEGG analysis of the DEGs in the leaves (a) and tubers (b) of the Qs9 plants, or the leaves (c) and tubers (d) of the D187 plants under the heat-stress treatment of different plant parts (affecting tuberization).

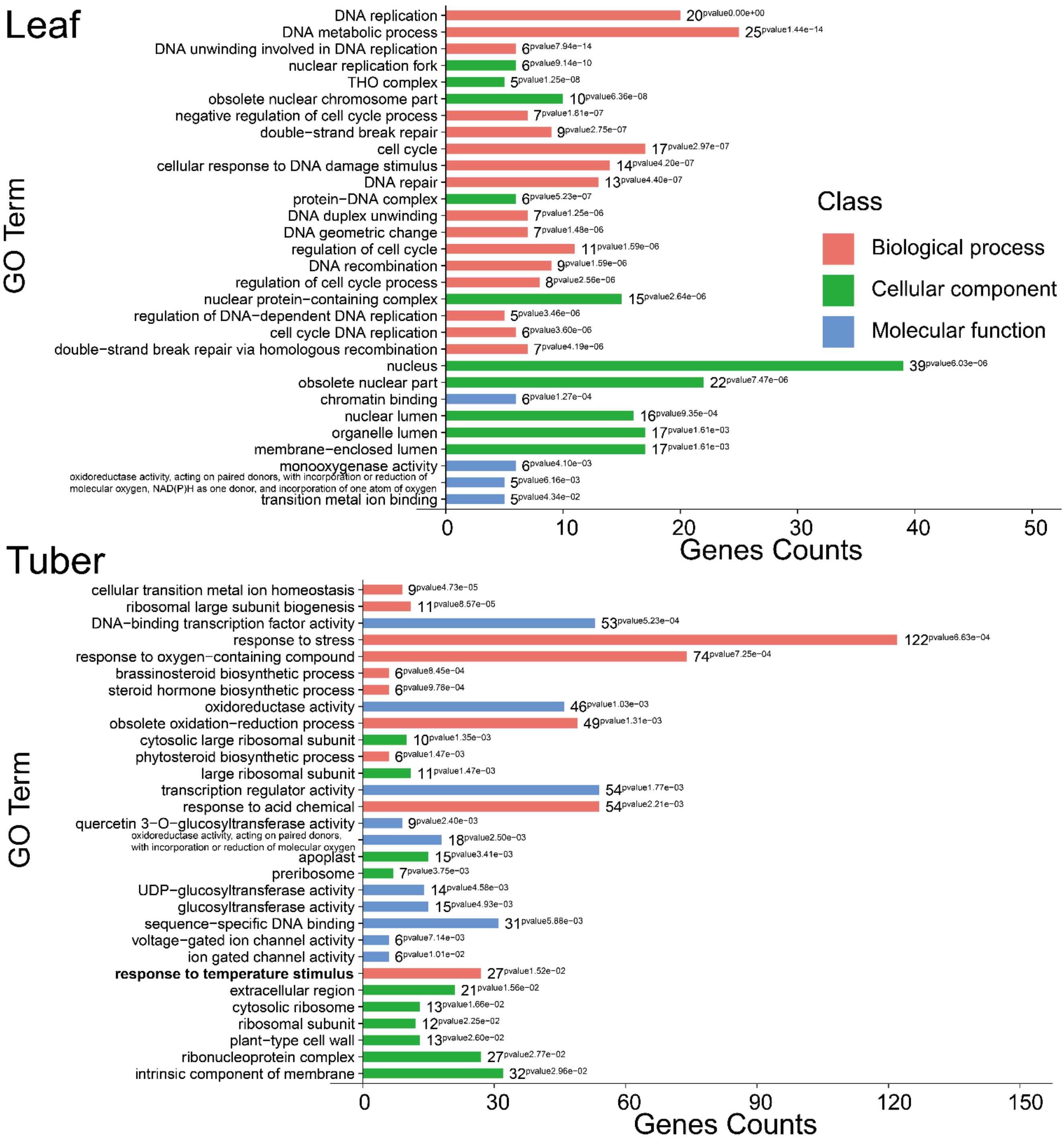

Figure S5. GO analysis of the DEGs in the leaves and tubers of the Qs9 plants under the heat-stress treatment of different plant parts (not affecting tuberization).

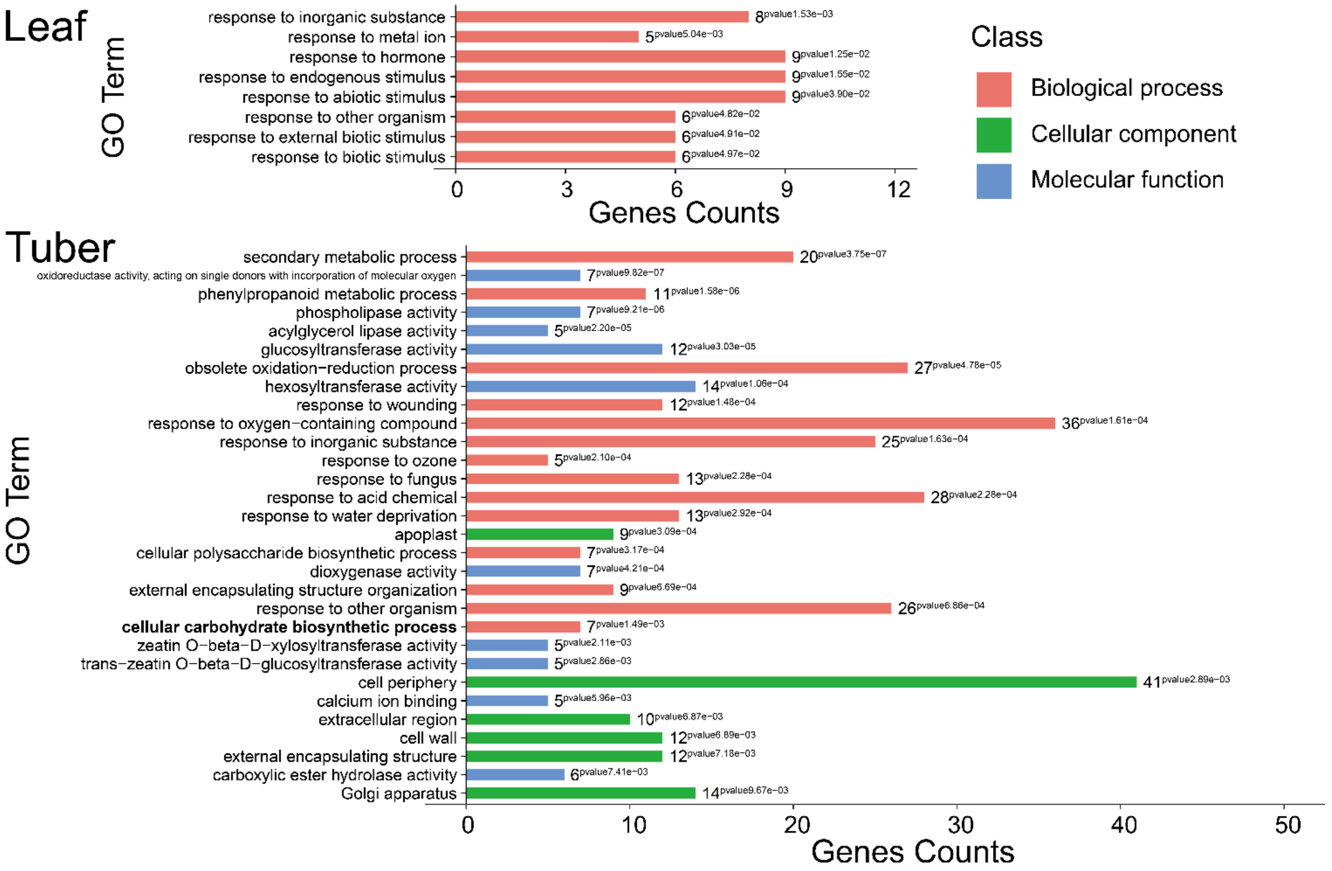

Figure S6. GO analysis of the DEGs in the leaves and tubers of the D187 plants under the heat-stress treatment of different plant parts (not affecting tuberization).

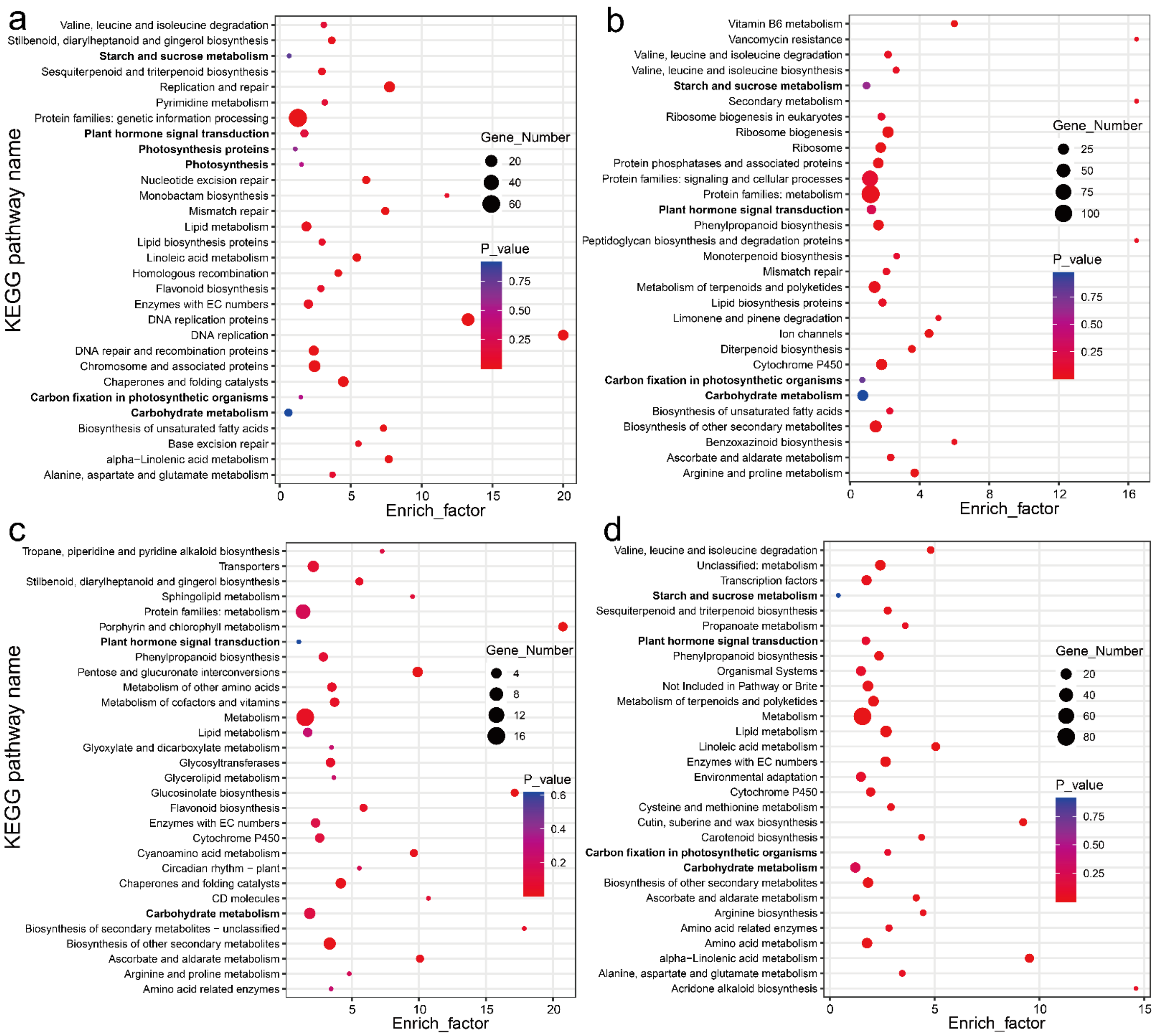

Figure S7. KEGG analysis of the DEGs in the leaves (a) and tubers (b) of the Qs9, or the leaves (c) and tubers (d) of the D187 plants under the heat-stress treatment of different plant parts (not affecting tuberization).

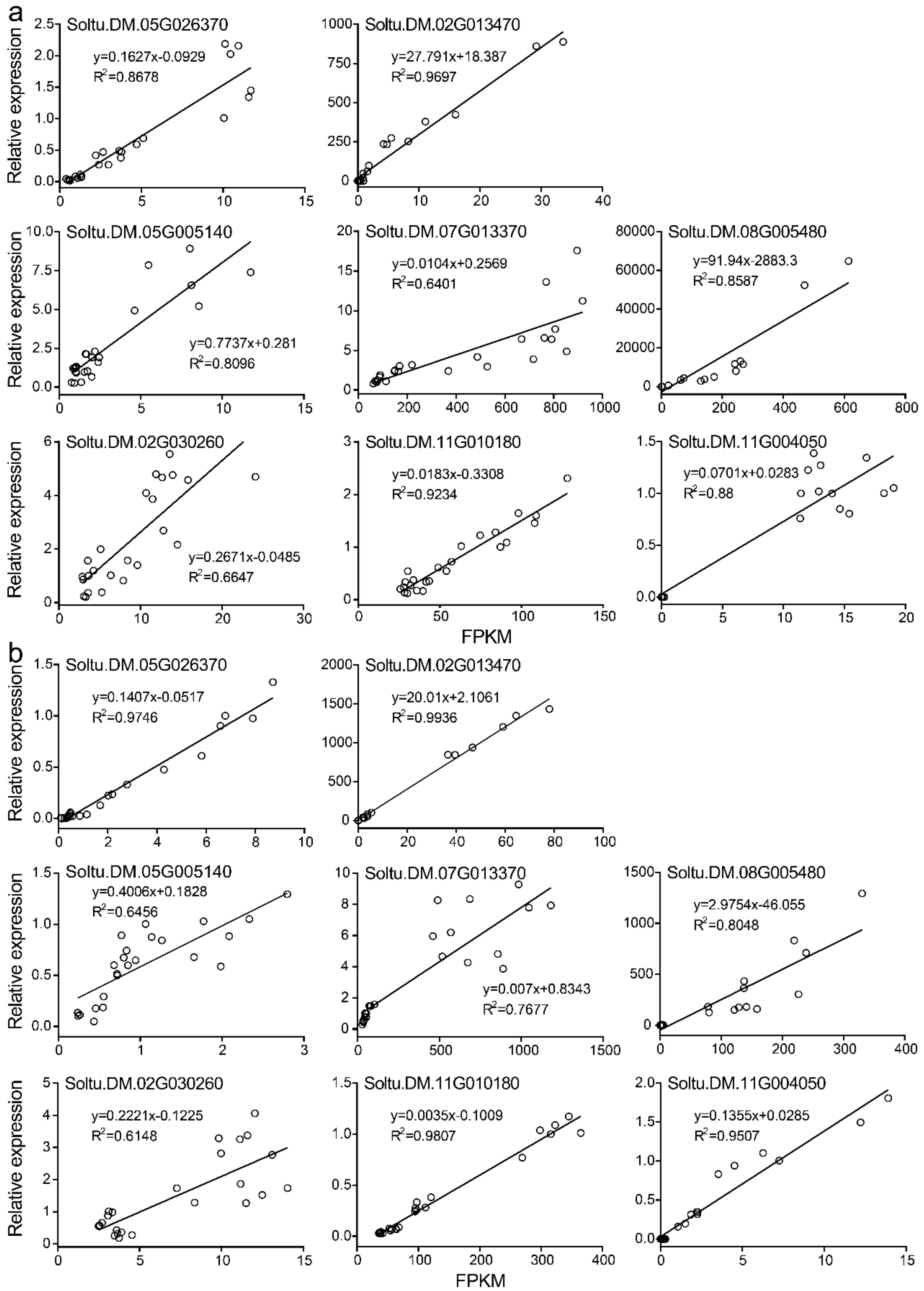

Figure S8. The linear correlation between the gene expression, via RT–qPCR (y-axis), from the Qs9 (a) and D187 (b) plants on one hand and the FPKM obtained from RNA-seq (x-axis).
